# A Pilot Study to Examine the Effect of Chronic Treatment with Immunosuppressive Drugs on Mucociliary Clearance in a Vagotomized Murine Model

**DOI:** 10.1371/journal.pone.0045312

**Published:** 2012-09-20

**Authors:** Abhiram R. Bhashyam, Peter J. Mogayzel, Sharon McGrath-Morrow, Enid Neptune, Alla Malinina, James Fox, Beth L. Laube

**Affiliations:** 1 Department of Pediatrics, The Johns Hopkins Medical Institutions, Baltimore, Maryland, United States of America; 2 Department of Biomedical Engineering, The Johns Hopkins Medical Institutions, Baltimore, Maryland, United States of America; 3 Department of Medicine, The Johns Hopkins Medical Institutions, Baltimore, Maryland, United States of America; 4 Department of Radiology, The Johns Hopkins Medical Institutions, Baltimore, Maryland, United States of America; Universidade de Sao Paulo, Brazil

## Abstract

**Background:**

Previously, we have demonstrated that mucociliary clearance (MCC) is diminished within the first months after surgery in lung transplant patients and the explanation for the reduction in MCC is unknown. We hypothesized that chronic treatment with a commonly prescribed regimen of immunosuppressive drugs significantly impairs MCC. We tested this hypothesis in a murine model of lung transplantation.

**Methods:**

Fifteen C57BL/6 mice underwent vagotomy on the right side to simulate denervation associated with lung transplantation in humans. For 6 days, seven mice (controls) were intraperitoneally injected with three 100 µL doses of phosphate buffered saline and eight mice (immunosuppressed) were injected with three 100 µL injections of tacrolimus (1 mg/kg), mycophenolate mofetil (30 mg/kg), and prednisone (2 mg/kg) once daily. Then, mice inhaled the radioisotope ^99m^technetium and underwent gamma camera imaging of their lungs for 6.5 hrs. Counts in the right lung at 1–1.5 hrs and at 6–6.5 hrs were first background-corrected and then decay-corrected to time 0 counts. Decay-corrected counts were then divided by time 0 counts. Retention at each time point was subtracted from 1.00 and multiplied by 100% to obtain percent removed by mucociliary clearance.

**Results:**

Although there was a slowing of MCC at 1–1.5 hrs for the immunosuppressed mice, there was no statistical difference in MCC measured at 1–1.5 hrs for the two groups of mice. At 6–6.5 hrs, MCC was significantly slower in the immunosuppressed mice, compared to controls, with 7.78±5.9% cleared versus 23.01±11.7% cleared, respectively (p = 0.006).

**Conclusions:**

These preliminary results suggest that chronic treatment with immunosuppressive medications significantly slows MCC in vagotomized C57BL/6 mice. These findings could shed light on why MCC is reduced in lung transplant patients whose lungs are denervated during surgery and who are chronically treated with immunosuppressive drugs post surgery.

## Introduction

Mucociliary clearance (MCC) is a vital pulmonary defense mechanism providing protection in the lower and upper airways against inhaled pathogens and irritants. Insoluble agents that deposit in the airway mucus are removed within hours by the MCC apparatus. Impairment to this defense mechanism leads to prolonged exposure to irritants, bacteria and viruses, the development of infections and subsequent re-injury to the MCC apparatus [1].

Previously, we have demonstrated that MCC is diminished within the first months after surgery in lung transplant patients and does not improve for at least 12 months thereafter [2]. There are several aspects of the lung transplantation process that might account for the observed reduction in MCC in patients. Lung transplantation requires a period of anesthesia with volatile agents such as isoflurane. Similar agents (halothane and enflurane) have been shown to impair MCC in the short-term (hours) in canine models [3]–[4]. However, the depressant effects of these volatile anesthetic agents on cilia [5] and on tracheal flow appear to be reversible [4]. This reversibility suggests that mucus clearance would be restored post-operatively in the absence of additional factors. Devascularization, as measured by ischemic time, may be another causative factor of the observed reduction in MCC following lung transplantation. However, results from a previous study in our laboratory showed that ischemic time was not associated with a reduction in MCC in the transplanted lung of individuals who had undergone transplantation [2]. The reduction in MCC also could be due to loss of vagal control in the lungs. During transplantation, the lung is denervated and the nerves are not reconnected. Nevertheless, we found in a previous study in mice that denervation did not affect basal MCC, suggesting that denervation alone does not account for the observed reduction in MCC in lung transplant patients [6]. The reduction in MCC also could be the result of treatment with immunosuppressive drugs to prevent rejection. The most commonly prescribed combination of drugs includes tacrolimus, mycophenolate mofetil (MMF), and prednisone [7]. Some of these drugs are known to alter Ca^2+^ fluxes, which have direct effects on ciliary beat frequency and MCC [8]–[9].

In this pilot study, we hypothesized that chronic treatment with this common regimen of immunosuppressive drugs would significantly impair MCC in mice that are unilaterally vagotomized, compared to placebo treated mice. If this combination reduces MCC in a mouse model, it could have important translational implications toward understanding the mechanism behind reduced clearance in patients who undergo lung transplantation.

## Materials and Methods

### Ethics Statement

Experiments were performed according to animal protocol MO07M147, which was approved by the Animal Care Use Committee of the Johns Hopkins University School of Medicine.

### Mouse Cohorts

Mucociliary clearance was compared in 8–10 week old, male C57BL/6 mice that were unilaterally vagotomized on the right side and either treated with the three-drug cocktail of immunosuppression medications, or placebo. Animals were purchased from Jackson Laboratories (Bar Harbor, ME).

### Vagotomy

Vagotomy was accomplished by transection of the right vagus, as described previously [6]. Prior to surgery, mice were weighed and anesthetized intraperitoneally with 100 µg/g ketamine and 16 µg/g xylazine. While anesthetized, a medial cervical incision was made and a 1–2 cm portion of the vagus was exposed, excised, and ligated such that a >0.5 cm gap was established between the proximal and distal nerve stumps. The nerve stumps were tightly ligated to prevent reanastomosis of the vagus during the recovery period. Mucociliary clearance was measured 7 days after this surgery and the gap between the nerve stumps was in effect at the time of those measurements.

### Treatment

Mice were divided into either control, or immunosuppressed groups. Eight mice (immunosuppressed) received doses of tacrolimus (Prograf, Astellas Pharma US, Inc., Deerfield, IL) (1 mg/kg), MMF (CellCept, Roche Pharmaceuticals, Boulder, CO) (30 mg/kg), and prednisone (2 mg/kg) dissolved in PBS/1% DMSO in 3 separate 100 µL intraperitoneal (i.p.) injections. In previous protocols using murine models, other investigators have shown that i.p. injection of these dosages results in positive immunosuppressive effects with limited toxicity [10]–[12].

Drugs were combined and administered as a cocktail instead of administering each individually. This is because previous studies have shown that drug interactions in combination delivery are not equivalent to the effects of the linear addition of the combination’s constituents [13].

Seven mice served as controls and received three separate 100 µL i.p. injections of PBS/1% DMSO solution. All mice were vagotomized on day 1 and given daily treatment for 6 days. MCC was quantified 7 days after vagotomy.

As a gauge of general health during the treatment period, each mouse was weighed daily and the final percent weight difference between day 1 and 7 was compared for the two groups.

### Oropharyngeal Aspiration

Mucociliary clearance was quantified using a non-invasive, oropharyngeal aspiration procedure described previously [14]–[15]. Both groups of mice were anesthetized by i.p. injection of ketamine (100 µg/g) and xylazine (16 µg/g) and suspended from their upper incisors at a 45° incline. Then, one 50 µl aliquot of normal saline containing 50–70 µCi of the radioisotope ^99m^technetium-labeled sulfur colloid (^99m^Tc-SC) was introduced into the distal part of the oropharynx and aspirated.

### Gamma Scintigraphy Imaging Procedure

Mouse lungs were imaged immediately after aspiration (time 0) and at 1–1.5 hrs and 6–6.5 hrs, thereafter, using an X-SPECT gamma camera (Gamma Medica, Inc. Northridge, CA) with pinhole collimation.

The effects of the anesthesia lasted approximately 45 min to 1 hr, so all mice were awake for most of the time between the first and subsequent imaging procedures. Both groups of mice were re-anesthetized a few minutes before the 1–1.5 hr and the 6–6.5 hr image was obtained. Only a quarter of the original dose was used at the 1–1.5 hr time point, since the mice had not fully recovered from the time 0 anesthetic prior to imaging.

We analyzed images from the 1–1.5 hr and the 6–6.5 hr time points because the first phase of mucociliary clearance of insoluble particles from the respiratory tract (clearance from ciliated airways) continues for between 20–24 hrs in animals and man, with half-times between 3 and 12 hrs [14], [16]–[17]. Based on this information, we reasoned that we would be able to detect the greatest signal in terms of MCC from the ciliated airways by quantifying MCC over approximately 6 hrs.

### Quantification of Mucociliary Clearance

Counts in the right lung at 1–1.5 hrs and at 6–6.5 hrs were first background-corrected and then decay-corrected to time 0 counts. Decay-corrected counts were then divided by time 0 counts. Retention at each time point was subtracted from 1.00 and multiplied by 100% to obtain percent removed by mucociliary clearance. Equations (1) and (2) summarize how MCC was determined:
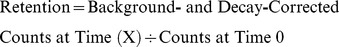
(1)

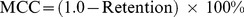
(2)


The left lung was not included in the analysis because interference/noise from the gastrointestinal tract made it difficult to acquire meaningful counts from the left lung [6].

### Quantification of Aerosol Distribution

**Figure 1:**
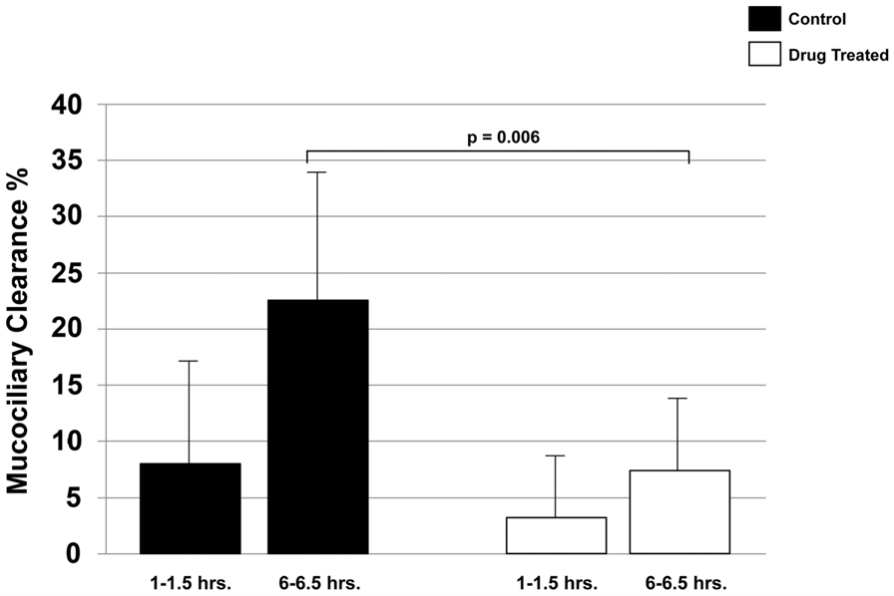
Mean MCC (±SD) from the right lung between 1–1.5 hours and 6–6.5 hours in 7 C57BL/6 control mice (dark bar) and 8 drug-treated C57BL/6 mice (light bars). There was a trend toward slower clearance in the drug-treated mice, compared to controls, at 1–1.5 hours, but differences in MCC were not statistically significant. Mucociliary clearance was statistically significantly slower in the drug-treated mice, compared to controls, at 6–6.5 hours (p = 0.006).

It is well known that the site of deposition of the radiotracer can affect MCC. For example, insoluble radiolabeled particles that deposit in ciliated airways will be cleared from the respiratory tract with half-times between 3–12 hrs in both animals and man [14], [16]–[17]. Removal from non-ciliated lung regions does not usually occur by MCC mechanisms and requires more than 24 hrs. To avoid the possibility of differences in MCC due to differences in regional lung deposition, we analyzed the initial aerosol distribution within the right lung of all mice in this study. A detailed explanation as to how distribution was determined is found in Bhashyam et al. [6]. Briefly, the right lung image of each animal was divided into 9 smaller squares. The square that was most central and closest to the trachea was identified as the central region (C). The remaining squares were collectively identified as the peripheral region (P). See Figure 1, Bhashyam et al. [6]. Distribution was quantified in terms of a central to peripheral ratio (C:P ratio), as described previously [6]. High C:P ratios indicated greater deposition of the radiotracer in the large airways. Low C:P ratios indicated greater deposition in the smaller airways and alveoli. This method of quantifying aerosol deposition within the lung has its limitations. This is mainly due to the fact that large airways overlap smaller airways and alveoli in this 2D representation. Nevertheless, based on anatomy, we assumed that the central region in the 2D image contained more large airways than smaller airways and the peripheral region contained more small airways and alveoli than large airways.

### Qualitative Analyses of Histological Changes in Lung

Qualitative histological evaluations were performed to identify possible differences in treated and untreated lungs that could account for the observed changes in MCC. A separate cohort of five immunosuppressed mice and five control mice were euthanized and the excised lungs and trachea were inflated with warmed (50–55°C) 1% low melt agarose at 30 cm H_2_O, as described previously [15]. The inflation pressure was measured continuously until the agarose started to gel. Lungs were fixed overnight in 4% paraformaldehyde. The lung and trachea were then paraffin-embedded, cut into five-micron sections and stained with hematoxylin and eosin (H&E). H&E sections of trachea and large bronchi were qualitatively evaluated for the presence of ciliated epithelial cells at 100 X and 40 X magnification.

**Figure 2:**
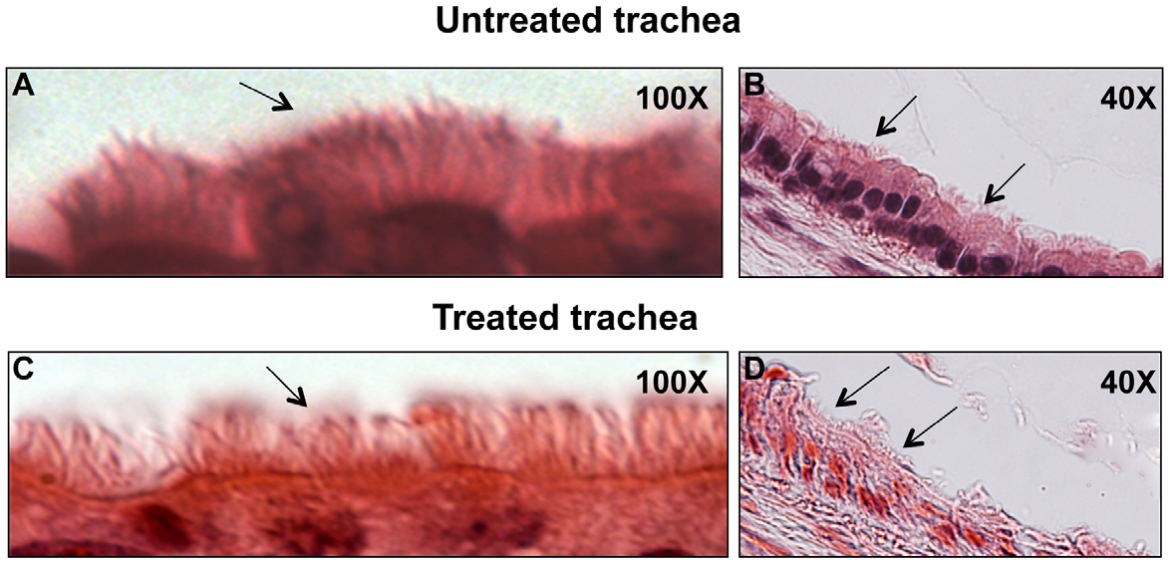
Representative examples of ciliated epithelial cells in the trachea of control and drug-treated mice. Tissue was evaluated at 100 X and at 40 X magnification for the control (A and B) and drug-treated mice (C and D), respectively. Black arrows point to cilia. Qualitatively, the presence of ciliated epithelial cells in the airways in each of the representive animals is similar.

Immunohistochemistry was performed on mouse lung tissue to determine possible differences in PAS and iNOS expression. Sections were prepared from the same separate cohort of five immuonsuppressed mice and five control mice as described above under Qualitative Analysis of Histological Changes in Lung.

#### PAS Expression

Formalin-fixed lungs were embedded in paraffin and sectioned to five microns. The tissue was deparaffinized and hydrated before staining. Sections were stained in 0.5% Periodic Acid and Schiff's Reagent for 5 minutes and 15 minutes, respectively. After counterstaining with hematoxylin, sections were rinsed with water, dehydrated and mounted. Prepared sections were qualitatively evaluated at 20X magnification for PAS expression.

#### iNOS Expression

Lung sections were deparaffinized and rehydrated with water. Antigens were retrieved by heating the sections in BorgDecloaketRTU (Biocare Medical; Concord, CA) for 10 min. at 95°C. After antigen retrieval, non-specific binding was blocked using Ultra V Block (UltraVision Detection System from Thermo Scientific; Fremont, CA) for 10 min. Then, slides were incubated in a humidity chamber with primary antibody from BD Bioscience (San Jose, CA) at 1∶400 dilution for 1 hr. at room temperature. As negative controls, adjacent slides were incubated in normal rabbit serum. After two buffer washes, slides were incubated for 10 min. with biotinylated Goat Snti-polyvalent (UltraVision Detection System), followed by a buffer wash and incubated for 10 min. in Streptavidin Peroxidase (UltraVision Detection System). After a buffer wash, staining of interest was developed with DAB chromagen (Dako; Carpinteria, CA) and counterstained with Fast Green (Sigma; St.Louis, MO). Slides were then dehydrated and mounted with Cytoseal (Thermo Scientific). Prepared sections were qualitatively evaluated at 20X magnification for iNOS expression.

### Statistical Methods

Data are presented as mean ± standard deviation for MCC from the right lung. A non-parametric, two-sided Mann-Whitney U test was used to compare MCC at 1–1.5 hrs, MCC at 6–6.5 hrs, C:P ratio, and percent weight change in the control and immunosuppressed groups of mice. P-values ≤0.05 indicated a statistically significant difference between comparisons. Pair-wise comparisons were performed using the Statistics Toolbox in MATLAB version 7.7.0 (The Mathworks, Inc., Natick, MA).

## Results

### Effect of Immunosuppression on Body Weight

Before surgery, control animals weighed 25.74±2.11 grams and immunosuppressed animals weighed 24.10±1.76 grams. Following treatment, mice in both groups had lost weight. On day 7, controls weighed 23.84±2.83 grams and immunosuppressed animals weighed 21.94±1.15 grams. However, there was no statistically significant difference between the groups, with percent change in body weight averaging −8±5% and −9±6% in the control and immunosuppressed mice, respectively.

### Effect of Immunosuppression on MCC

At 1–1.5 hrs post aspiration, MCC from the right lung of the immunosuppressed mice averaged 3.81±5.66%, which was not statistically different from control mice with 7.58±9.20% (Figure 1). By 6–6.5 hours post aspiration, MCC from the right lung was statistically significantly reduced in the immunosuppressed animals compared to control animals, averaging 7.78±5.9% and 23.01±11.7%, for the two groups, respectively (p = 0.006) (Figure 1).

### Distribution of Radiotracer: C:P Ratio

Regional distribution of radiotracer within the right lung was not statistically different for the two groups of mice. The C:P ratio averaged 1.60±0.36 and 2.26±0.90 in control and immunosuppressed mice, respectively (p = 0.105).

### Effect of Immunosuppression on Lung Histology

H&E sections from the trachea of a control and immunosuppressed mouse are shown in Figure 2. Qualitatively, there was no difference in the presence of ciliated epithelial cells observed in the H&E sections of the trachea at either 100 X, or 40 X, magnification for the control (Figures 2A and 2B) and the drug-treated mice (Figures 2C and 2D), respectively. The presence of cilia decreased significantly in the H&E sections for the bronchi at both magnifications in the control and drug-treated mice (sections not shown), making a qualitative comparison impossible.

#### PAS Expression

**Figure 3:**
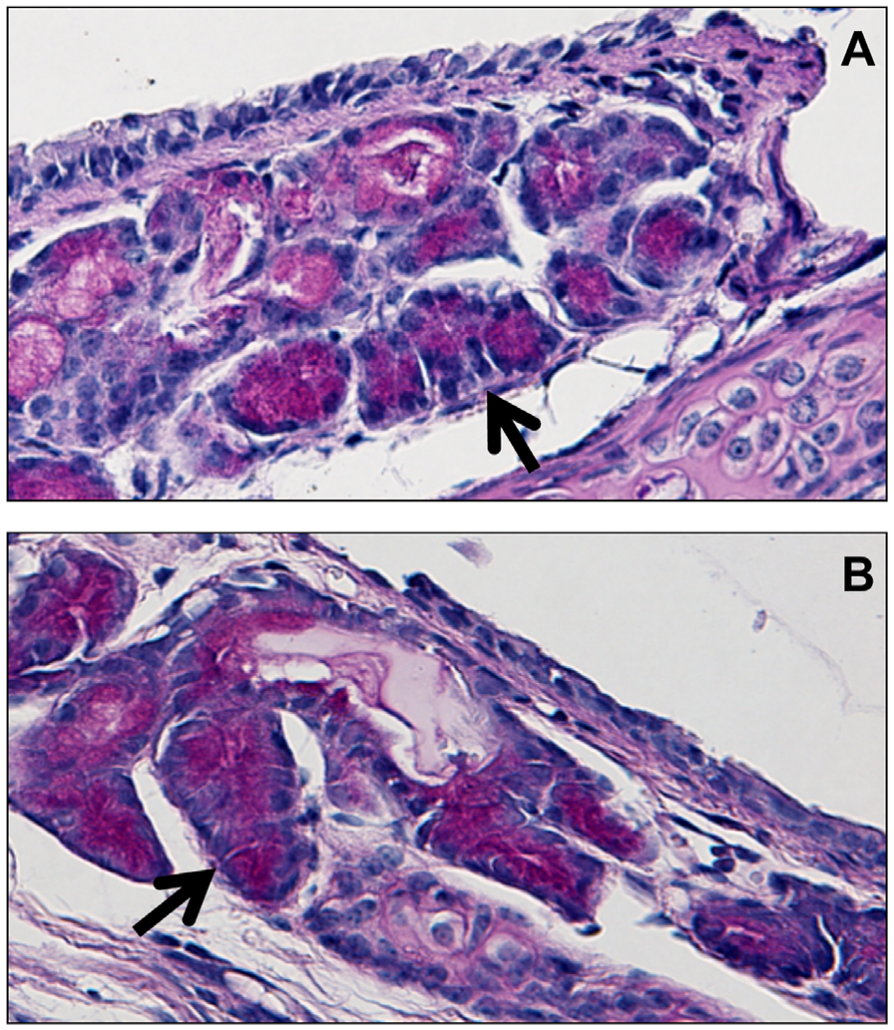
PAS staining of seromucous cells in tracheal glands of vagotomized mice. **A.** Trachea of mouse treated with immunosuppressive agents. **B.** Trachea of mouse not treated with immunosuppressive agents. Black arrows point to PAS staining in seromucous cells. Qualitatively, both the drug-treated animals and control mice appeared to have similar PAS staining in the seromucous cells of the tracheal glands.

Qualitatively, both the drug-treated animals and control mice appeared to have similar PAS staining in the seromucous cells of the tracheal glands (Figure 3), suggesting that these cells remained in tact despite chronic treatment.

#### iNOS Expression

**Figure 4:**
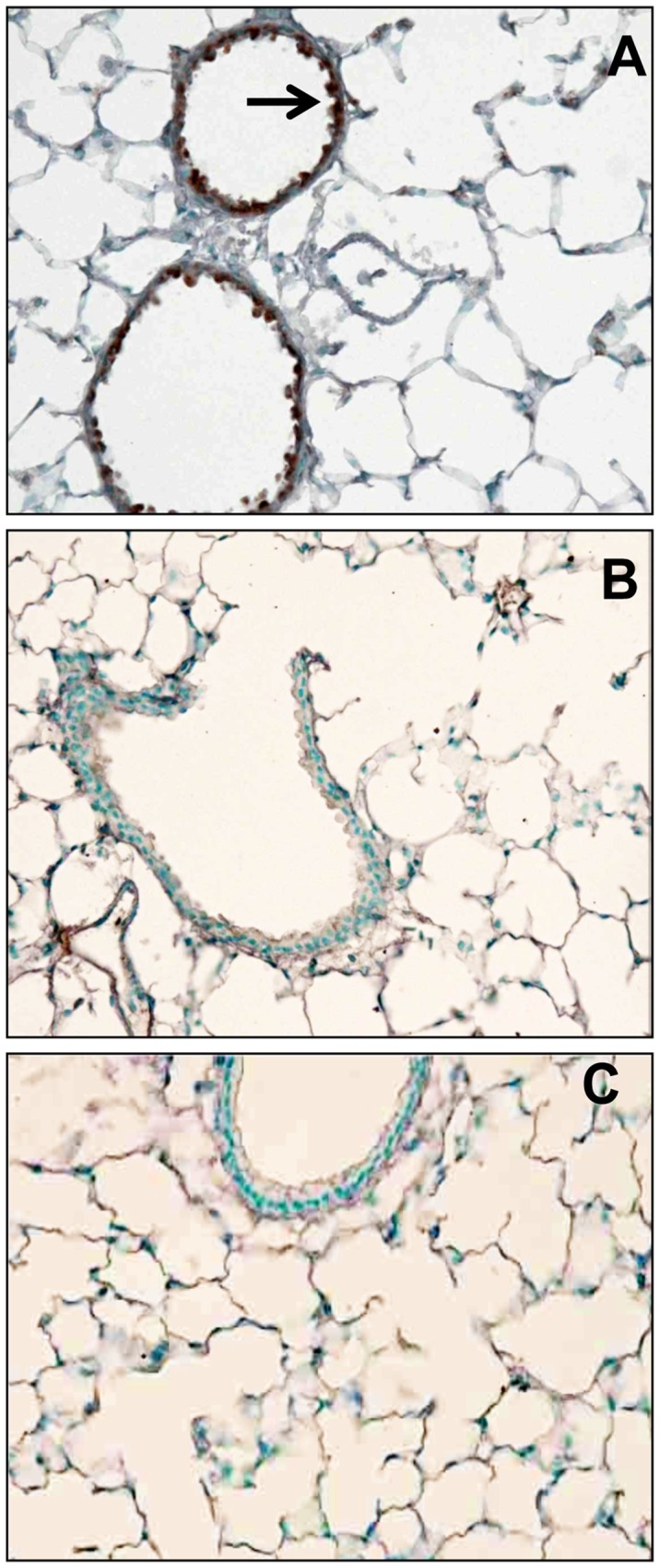
iNOS staining in airways of vagotomized mice. **A.** Increased iNOS staining (brown) in bronchial epithelium of adult mouse treated with immunosuppressive agents (Black arrow). iNOS staining was qualitatively increased in the bronchial epithelium of five of the five drug-treated mice. **B.** Minimal iNOS staining in airway of adult mouse that was not treated with immunosuppressive agents. Minimal iNOS staining was observed in five of the five mice not treated with immunosuppressive agents. **C.** No first antibody control.

iNOS staining was qualitatively increased in the bronchial epithelium of five of the five drug-treated mice (Figure 4). Minimal iNOS staining was observed in five of the five mice that were not treated with immunosuppressive agents (Figure 4).

## Discussion

This study was designed as a pilot study to answer a basic question: Does one of the most common immunosuppressive drug combinations that is currently administered to lung transplant patients have a detrimental effect on mucociliary clearance? In a previous study in lung transplant patients, we reported that MCC is slowed after surgery and remains impaired for at least one-year, thereafter [2]. Although the mechanism for the impairment is unknown, we hypothesized that chronic treatment with commonly prescribed immunosuppressive drugs could be one explanation. We tested this hypothesis in a vagotomized murine model. At 1–1.5 hrs after inhalation of the radioisotopic marker, there was a trend towards slower clearance in the drug-treated mice, compared to controls. However, those differences in MCC were not statistically significant. By 6–6.5 hrs, MCC was statistically significantly slower in the immunosuppressed mice, compared to controls. The lack of statistical significance in MCC at 1–1.5 hrs was likely due to greater variability in the measurement at that time point. The coefficients of variation for MCC in the immunosuppressed and control mice at 1–1.5 hrs were 1.49 and 1.21, respectively. This is in contrast to the much lower coefficients of variation observed at 6–6.5 hrs, with 0.76 and 0.51, respectively. It is unknown why there was greater variability in the MCC measurements after 1–1.5 hrs, compared to 6–6.5 hrs.

We chose not to include non-vagotomized animals in these studies because human lungs are denervated following transplantation and denervated mice chronically treated with immunosuppressive drugs seemed the best model for translating our findings to humans, which is our goal. Our findings do not rule out the possibility that treatment with immunosuppressive drugs could affect MCC in non-denervated animals and in humans who have not undergone lung transplantation [18]–[19]. For this reason, a group of non-vagotomized mice would be interesting to look at in future studies in order to determine the potential impact of chronic immunosuppression on MCC in transplant populations that don’t undergo denervation (i.e. other solid organ transplantation patients).

Mice were treated with a cocktail of tacrolimus, which inhibits calcineurin, MMF, which impairs B- and T-cell proliferation, and prednisone, which prevents acute rejection [7]. These drugs were chosen to emulate one of the most common regimens currently used to treat lung transplantation patients. Doses for each of the drugs were based on previous efficacy in terms of reducing, or eliminating, acute rejection in other murine models [10]–[12].

In terms of translating the doses used in this murine model to doses used to treat patients after lung transplantation, clinical doses for MMF range between 1 and 1.5 g daily and prednisone ranges between 250–1000 mg daily [20]. Assuming an average human weight of 70 kg, the doses of MMF and prednisone that were administered in our study are similar to clinical doses for patients who have undergone transplantation. The dose of tacrolimus that was used in this murine model was significantly higher than the dose used in humans, which ranges between 0.1–0.2 mg/kg [20]. We used a higher dose of tacrolimus because previous investigators have shown that dosages of 0.5 mg/kg or lower are sub-therapeutic in mice, while higher doses (i.e. 2 mg/kg) are therapeutic based on trough serum levels [10].

One explanation for the reduced MCC in the immunosuppressed mice could be a reduction in mucus transport due to a loss of ciliated epithelial cells. Molecular mechanisms associated with prednisone and MMF have been shown to affect epithelial integrity [21]. However, qualitative histological examination of the airway sections, as described, did not reveal any difference in the presence of ciliated epithelial cells in the trachea of the immunosuppressed mice, compared to controls (Figure 2A–D). These results should not be surprising since, contrary to what is found in other species, the majority (50–60%) of cells in murine airway epithelia is non-ciliated Clara cells [22] and ciliated cells often occur in scattered patches [23]. Thus, there may be too few ciliated cells to observe differences based on a qualitative analysis. Other, more quantitative, histological analyses that were beyond the scope of this pilot study might reveal differences.

PAS staining results suggested intact seromucous cells in the tracheal glands of the chronically drug-treated animals (Figure 3). It is unknown if there were any differences between other mucous producing cells, since these analyses were performed retrospectively and were not part of the original study design.

It is interesting that, qualitatively, the airways of the five drug-treated animals showed significantly greater amounts of iNOS than the five control animals (Figure 4). The explanation for this difference is unknown. However, iNOS can be induced in macrophages in response to stress such as drug toxicity or infection [24] and, in our model, it appears that increased iNOS is associated with impaired MCC. Nitric oxide (NO) levels were not quantified in these animals. Therefore, it is not known if this histological finding of an increase in iNOS in the airways of the treated animals was associated with increased levels of NO. Future studies could further examine the relationship between increased iNOS and decreased MCC in this model.

Daily administration of the immunosuppression drug cocktail may have altered the general health of the drug-treated mice and this may have affected MCC in some unknown way. However, we were unable to discern any difference in the overall health of the two groups of mice. The drug-treated animals did not appear lethargic and they did not lose more weight than the control animals.

Differences in the initial deposition of the radioisotope such that the marker was systematically deposited in more proximal airways in the control mice and more distally in the immunosuppressed animals might account for the faster MCC observed at 6–6.5 hrs in the control group. However, analysis of the deposition distribution in each of the mice showed it trending in the opposite direction. C:P ratios were higher in the immunosuppressed mice, compared to control mice. Since higher C:P ratios indicate more proximal deposition of the radiomarker, this trend should have resulted in an increase in MCC in the immunosuppressed mice, compared to controls. However, this was not the case. Thus, it is unlikely that a systematic difference in the distribution of the radiolabeled aerosol particles was responsible for the observed differences in MCC for the two groups of animals.

Another possible explanation for our observation of depressed MCC in the vagotomized, immunosuppressed mice is the combined negative molecular effects of tacrolimus, MMF and prednisone. Molecular control of MCC is an intricate process that is well-described in a recent review by Salathe [9]. In particular, changes in baseline calcium ion concentration can modulate ciliary beat frequency (CBF), which in turn can increase, or decrease, MCC [9]. Immunosuppressive medications like tacrolimus have the potential to alter this ionic concentration. Tacrolimus suppresses the FKBP-4 gene which produces the FK506 binding protein (FKBP) in human cell cultures. As a result, lowered FKBP levels inhibit Ca^++^ oscillations, which experimentally reduce CBF [25]. In addition, tacrolimus decreases Ca^++^ content in thapsigargin-sensitive stores, suggesting that even partial depletion of Ca^++^ levels causes an inhibition of Ca^++^ oscillations that could reduce CBF and, consequently, MCC [8], [25]. Future studies that determine the oscillations in the Ca++ influx, the levels of CBF, the expression of the FKBP-4 gene, as well as the levels of FKBP and cGMP in the lungs of these animals could provide much needed insight as to why MCC is depressed in these vagotomized, immunosuppressed mice.

Prednisone, another agent used in this experiment, could also depress MCC in our mouse model. Working with patients with sarcoidosis, Hasani et al. [26] demonstrated that patients who were using inhaled corticosteroids demonstrated a greater degree of impairment in mucociliary clearance than patients who were either in remission, or were receiving oral corticosteroid therapy. They concluded that their findings raised the question of possible consequences of long-term inhaled immunosuppressive therapy on mucociliary clearance.

It was beyond the scope of this study to examine the individual effects of the drugs that make up the typical immunosuppressive drug cocktail on mucus clearance in our mouse model mice. Future studies in which the three drugs are administered separately, or in alternate combinations, could determine if there are differences between the drugs, or drug combinations, in terms of their effect on MCC, and if one drug is more potent than another in reducing MCC in this animal model.

The notion that immunosuppressive drugs may have impaired CBF in the mice in these studies is supported by our previous findings in humans who underwent lung transplantation. In those studies, we found that MCC in the transplanted lung was significantly reduced after surgery and treatment with immunosuppressive drugs. However, the impairment was not permanent. Mucociliary clearance was immediately restored to normal levels following inhalation of 4 puffs of the beta_2_ adrenergic-agonist albuterol from a pressurized metered dose inhaler [2]. These findings suggest that lung transplantation leaves the cilia functional and intact, but the immunosuppressive drugs inhibit or reduce the Ca++ signal and, thereby, reduce CBF. In humans, this inhibition of CBF appears to be overcome with inhalation of a beta_2_ adrenergic-agonist. It was beyond the scope of these experiments to determine if a beta_2_ adrenergic-agonist would also rescue MCC in the immunosuppressed mice.

### Conclusions

Results from these experiments indicate that chronic treatment with a commonly used cocktail of immunosuppressive medications significantly slows MCC in vagotomized C57BL/6 mice. These findings could shed light on why MCC is slowed in lung transplant patients, as we have reported previously (2), since these patients are chronically treated with similar immunosuppressive drugs post surgery. Future studies are needed to determine if one drug is more potent than another in reducing MCC in this animal model.
